# Supplementary material for: Patterns of Intron Gain and Loss in Fungi
Source: PLoS Biol. 2004 Nov 30;2(12):e422. doi: 10.1371/journal.pbio.0020422 (PMC532390; doi:10.1371/journal.pbio.0020422)
Supplement: Table S1 — Also available at http://genes.mit.edu/NielsenEtAl/. (4.3 MB ZIP). [file pbio.0020422.st001.zip › NielsenEtAl/html/388.html]

AN3456.1.NCU02430.1.MG03583.1.FG09855.1


```
 CLUSTAL W (1.82) Multiple Sequence Alignments - Introns Inserted


Sequence 1: NCU02430.1	542 aa
Sequence 2: MG03583.1	627 aa
Sequence 3: FG09855.1	597 aa
Sequence 4: AN3456.1	594 aa
Alignment Length: 636 aa
Number Identitical Residues: 210 aa
Alignment Score (without introns) 11460


MG03583.1 	MIEVELGASIPPDTAH0AVSVSLPTWQANVGYEEGQSWVVDRMTTGYPRFFIHK~TITGF
NCU02430.1	----------------~-------------------------------------~-----
FG09855.1 	MPVIGLGESIPSDTAH0AVSVSLPTWKANVGYEEGEDWVVNRMTTGYP-----R2GIQAF
AN3456.1  	-MLQEPGGPVPPNTDH0AVSVSLPTWEANVAYEEGELWLVNKMQCGYPRFFVHP~IIQEL
          	      . . ...:   : : : .: .:. . ....    .    . .            

MG03583.1 	ADELAARFGQPGQKA-MLFPNRQAALRCVDYVRGRAPSQSALAGLDAVHLTLTPEAPKVV
NCU02430.1	----------------MLFPTPTIASRCVDFIRSRAPAD-VCSNIEVVNLVLDMSDPE--
FG09855.1 	AKEIVDRYGSPGQQA-MLFPTPRVAKRCLDFIVQRISPE-LASQVRIFDFALDK--SKEL
AN3456.1  	AQEIVARFGNPEYEATTLFPSPNTARVCHTFILSRISRE-EAHKIRVLDFVPTK----EL
          	:..     ...  .:: ***.   *  *  ::  * . :     :  ..:.         

MG03583.1 	VDQLRPISPAVSAVLLSPEAFPVAKEYWQHSGDGVSSRRAEFCQNLFSAKLLVPEGSSPL
NCU02430.1	ARALEPLCPSISAVIMPQDGFPFAKQYWQHSGDGVSSRRAEFCHGLFKDGLLRPD-TELR
FG09855.1 	SPVLKKLTSTISAVLYPSDVFPVAKQYWQHTGEGTSSRRAEFCHGLFNDDLLIPAARTPP
AN3456.1  	ETELSTIQSLLSCVIYPKEHAKIAKQAWQHTGDGVSSRRGEFCLGALKDGLLTERKNVTG
          	   *  : . :*.*: . :   .**: ***:*:*.****.*** . :.  **        

MG03583.1 	SPQPPVFKSAKGPRRYRRETPSNDTPAPNGTNGATTPPKAAKTQEQESEEETERFLEERF
NCU02430.1	NAAVSAAKPCRGPKRYQRQASLD---AGGNQQTIMHGHIHRATGETAMIQETSRFLEERF
FG09855.1 	EAVQ--SRPFRGPRRYTRPGSID-----GVSSPVPTAPKRTSPENPVESIESSTFLEERF
AN3456.1  	DATR--QIIHKGPRRYQGKGSIN-------GIGKVSSHNAVSANKPHEGRESVQFIEERF
          	..        :**:**    . :                   . :     *:  *:****

MG03583.1 	GRNLDISLLPRARSAIKRRIAGAL-SADHDISSSHTFP--AMESNTRGVNNLVEDDVLIY
NCU02430.1	GRNLDLSFVHPAKSAIKRRIAGALRSADHDLGGSPSLSEKQMSSNTRGIANLREEDIYLF
FG09855.1 	GRNLDLSMVERAKSAIRRRIAGAM-AHEVDMNNGPLP---AMTSNTRGIPSLEETDVYLY
AN3456.1  	GRNLSTSLASKAKIAVRRRIAGVL-TADVELNEALEK-----ASQEGRVAGLSESDVFLF
          	****. *:   *: *::*****.: : : ::. .         *:   : .* * *: ::

MG03583.1 	PCGMNAIFHAHRLLMSAR----GRDLKSINFGFPYVDTLKILQKFGP-GCVFYGHASEAD
NCU02430.1	PCGMNAIFHAHRALYSIRTPPGSTPLKSVNFGFPYVDTLKILEKFNPSGALFYGHGSKSD
FG09855.1 	PAGMNAIFNAHRALLHAR-----EPAESINFGFPYVDTLKILQKFGP-GCLFYGHASPED
AN3456.1  	PSGMSSIFNSHRLLLEAR-----GSMKSVCFGFPYIDTLKILEKWGP-GCVFYGRGSSED
          	*.**.:**::** *   *        :*: *****:******:*:.* *.:***:.*  *

MG03583.1 	LDDLERRLEAGERYLALFCEFPGNPLLTCPNLARIRTMADKHDFAVVVDETIGTFSNINV
NCU02430.1	LDDLETRLESGERYLALFCEFPGNPLLTCPDLVRIRELADKYEFAVVVDETIGTFANVNV
FG09855.1 	LDDLEARLKKGERYLGLFCEFPGNPLLTCPDLTRIRKLADEYDFAIVIDETIGTFANINV
AN3456.1  	LNDLEARLAGGERFLALFTEFPGNPLLKTPDLKRIRALANKYEFAVVVDETVGNFLNINV
          	*:*** **  ***:*.** ********. *:* *** :*::::**:*:***:*.* *:**

MG03583.1 	LQYADIVVSSLTKIFSGDCNVMGGSSILNPNGRYYGALKAAVATDYEDTYWAEDVLFMER
NCU02430.1	LQFADIVVSSLTKIFSGDCNVMGGGAIFNPNSRYYSALKSFVQQQLEDTYWPEDVIFMER
FG09855.1 	LPVADIVVSSLTKIFSGDSNVMGGGLVLNPDSKYYRALKAAMEEIYEDNYWPEDVVFMER
AN3456.1  	LSHADIVVSSLTKIFSGDSNVMGGSAVLNPHGRYYPLLKDTFNRGYEDNLWVEDAVFLER
          	*  ***************.*****. ::**..:**  **  .    **. * **.:*:**

MG03583.1 	NSRDFASRVVRINVNAEAVCDLLHSHPLV~RRVHYPRVNADRANYEQCRLPSGGYGGLLS
NCU02430.1	NSRDFVARIDRVNANAEAICRVLQDHPLV~KTLYYPKYNDSRANYEAVKLPQGGYGGLIS
FG09855.1 	NSRDYESRVVRTNANSDAICEVLRNHHLV~KRIYYPKSNDSREHYEKVRLPDGGYGGLLS
AN3456.1  	NSRDFVSRIEKINHTTEVITAMLQESPIV1KNVYYPKFSPTLPFYEAFRTPNGGYGGLFS
          	****: :*: : * .::.:  :*:.  :* : ::**: .     **  : *.******:*

MG03583.1 	VVFRDKEAAVAFYDAIKTAKGPSLGTNFTLV~SPYVLLAHYNELDW~AASLGVDPNLLRI
NCU02430.1	VVLKGKKQAVAFYDAIETAKGPSLGTNFTLT~SPYVLLAHYQELDW~AEQYGVDRNLIRI
FG09855.1 	VVFQRKEYAQAFFDALPLAKGPSLGTNFTLV~SPYVLLAHYQELEW~AESFGVDPYLIRV
AN3456.1  	VTFHSTAEAIAFFDNLEVHKGPSLGTNFTLS2SPYTLLAHYSELDW0ASSFGVEFDLVRI
          	*.:: .  * **:* :   ***********  ***.*****.**:* * . **:  *:*:

MG03583.1 	SIGLGETEELVDTFKKALEVAEVAVGAAGKEEAPAAAPKVQ
NCU02430.1	SVGLEGTDELINVFTRALKVAEEQS------QYP-------
FG09855.1 	SVGLEDTTELGDIFTDALKAAEAVTVTG-------------
AN3456.1  	SVGLEDVPLLRAKVQQALDAVAKISK---------------
          	*:**  .  *   .  **...
```
